# Exploring challenges and community support for hard-to-reach persons with visual impairment in the Free State province, South Africa: A qualitative study

**DOI:** 10.4102/ajod.v15i0.1936

**Published:** 2026-05-29

**Authors:** Matieho B. Mokhua, Tuwani A. Rasengane, Joyce M. Tsoka-Gwegweni, Antor O. Ndep

**Affiliations:** 1 Department of Optometry, Faculty of Health Sciences, University of the Free State, Bloemfontein, South Africa; 2 Division of Public Health, Faculty of Health Sciences, University of the Free State, Bloemfontein, South Africa; 3 Department of Public Health, Faculty of Allied Medical Sciences, University of Calabar, Calabar, Nigeria

**Keywords:** mobilisation strategies, hard-to-reach, visual impairment, low vision, Free State province, South Africa

## Abstract

**Background:**

Visual impairment is the third most prevalent impairment worldwide. Persons with visual impairment may have to navigate complex societal barriers and marginalisation daily. Thus, making them hard to reach.

**Objectives:**

To explore mobilisation strategies for hard-to-reach persons with visual impairment among community health workers and low-vision clinicians in the Free State province, South Africa.

**Method:**

An exploratory qualitative approach was used to explore mobilisation strategies for hard-to-reach persons with visual impairment in the Free State province.

**Results:**

Two main themes were identified: (1) challenges of accessing eye care services and (2) social and community support. Affordability and accessibility of spectacles and low-vision devices remain significant challenges, serving as barriers to accessing eye care services, particularly in rural areas. Poor social and community support by local government structures of resources, such as funding of eye care and transport costs, may be available to support persons with visual impairment, but are not utilised effectively by the local government. Lack of physical infrastructure that is accessible to persons with visual impairment and the poor inclusion of persons with visual impairment in community programmes and decision-making processes to improve social interaction and foster independence are prevalent.

**Conclusion:**

The challenges limit the capacity to deliver appropriate interventions to persons with visual impairment, further exacerbating the gap in care for the hard-to-reach. This emphasises a need for a more equitable distribution of resources, increased funding for eye care services, and the employment of more specialised professionals in underserved areas.

**Contribution:**

Findings informed a set of recommendations for the benefit of hard-to-reach persons with visual impairment in the development of inclusive eye care policies and empowered both the hard-to-reach and community health care workers.

## Introduction

Visual impairment is the third leading impairment worldwide (James et al. 2018) and is recognised as a significant public health concern. Individuals with visual impairment experience many challenges, including poor social interaction (Klauke, Sondocie & Fine 2023), anxiety and depression (Demmin & Silverstein 2020). That is so because vision loss can lead to social isolation, reduced independence, communication barriers and diminished self-esteem, all of which increase vulnerability to mental health challenges (Marques et al. 2022) and reduced quality of life (Bonsaksen, Brunes & Heir 2025). Persons with visual impairment may experience daily marginalisation and societal barriers. When combined with poor social interaction and mental health difficulties, these factors can leave them isolated and harder to reach with essential services. The hard-to-reach persons with visual impairment require good social connections to overcome these challenges (Sri Takshara & Bhuvaneswari 2025). Social connections play a crucial role in the improvement of health outcomes and the mental well-being of individuals (Deitz et al. 2020). Building such a solid relationship requires community involvement to improve access to the required services needed by these individuals.

Various countries worldwide have implemented different strategies to reach hard-to-reach populations (Rockliffe et al. 2018). A strategy is generally understood as a structured plan of action that incorporates multiple approaches to achieve specific goals whilst considering both challenges and available resources (Modeste 1996). Studies have shown that well-designed strategies utilising a community-based approach, particularly in health promotion, have been effective in reaching older individuals from disadvantaged areas who are typically difficult to engage (Liljas et al. 2019). These improvements may be obtained through community health care workers (CHWs). Community health care workers are recognised as essential health providers within their local communities, delivering first-line care and support. Following the Alma-Ata Declaration over 40 years ago, many governments and non-governmental organisations adopted this model, which is now widely accepted worldwide (Rifkin 2016).

Despite facing various challenges, CHW programmes have demonstrated their effectiveness in improving community health (Werner et al. 2023). Community health care workers not only improve the health outcomes in communities, particularly for the marginalised, but they also build positive relationships between health facilities and communities (Ndambo et al. 2022), which in turn benefit even hard-to-reach persons with visual impairment. In Uganda, organising and implementing community eye clinics as part of community eye care mobilisation has been shown to enhance access to eye care services for hard-to-reach patients (Davey et al. 2020). However, there is limited evidence on the role of CHWs in providing eye care services in other sub-Saharan African countries (Faal et al. 2015). In this region, eye care services are primarily delivered by optometrists and ophthalmologists (Oduntan et al. 2015). Not all optometrists are regarded as low-vision clinicians (LVCS).

Low-vision clinicians offer rehabilitation services to persons with visual impairment, using optical and non-optical considerations (Shah et al. 2018). Low-vision clinicians and community health workers often operate in silos, which limits their ability to connect the large population of persons with visual impairment to appropriate care. South Africa faces several challenges, including limited resources, an uneven distribution of healthcare professionals between the public and private sectors, low skill levels, inadequate motivation, and a lack of managerial capacity in Africa (Centre for Social Development in Africa [CSDA] 2019). A key concern amongst these issues is the widening gap between the public and private health sectors, with the public sector serving the majority of the population and remaining a major area of focus (Productivity Commission 2009). This implies that a significant portion of individuals relying on the public healthcare sector in South Africa lack access to eye care, as it is not included in the country’s health promotion activity plan (Sithole 2017). This study therefore explored challenges and community support for hard-to-reach persons with visual impairment amongst CHWs and LVCS in the Free State province.

## Research methods and design

A descriptive exploratory qualitative research approach was used (Hunter, Mccallum & Howes 2019). Participants were selected purposively to ensure inclusion of specific groups with relevant experience, and the research questions associated with the study objectives were addressed. Participants included ten CHWs, five persons with visual impairment, and five LVCS who utilise the public health amenities sector in each of the five districts in the Free State province (Table 1).

**TABLE 1:** Characteristics of the community healthcare workers and low-vision clinician participants.

Participants	Qualification	Experience (years)
LVC 1	Bachelor’s degree	16–21
LVC 2	Bachelor’s degree	22–26
LVC 3	Bachelor’s degree	22–26
LVC 4	Master’s degree	16–21
LVC 5	Master’s degree	22–26
CHW 1	Grade 12	6–10
CHW 2	Grade 12	0–5
CHW 3	Grade 12	0–5
CHW 4	Grade 12	11–15
CHW 5	Grade 12	11–15
CHW 6	Grade 12	11–15
CHW 7	Grade 12	0–5
CHW 8	Grade 12	0–5
CHW 9	Grade 12	11–15
CHW 10	Grade 12	11–15
VIP 1	Higher certificate	0–5
VIP 2	Higher certificate	0–5
VIP 3	Grade 12	0–5
VIP 4	Bachelor’s degree	0–5
VIP 5	Higher certificate	6–10

LVC, low-vision clinicians; CHW, community health care workers; VIP, person with visual impairment.

### Ethical considerations

Ethical clearance to conduct this study was obtained from the University of the Free State Health Sciences Research Ethics Committee (HSREC) (No. UFS-HSD2024/1941/2801), and permission was obtained from the person responsible for managing the CHWs in the Free State province, as well as from persons with visual impairment and LVCS. The first author ensured that all participants completed the consent forms and shared information about the study. Signature guide (Typoscope) assisted all persons with visual impairment in signing the informed consent forms.

## Results

### Data collection

Data were collected in February 2025 using semi-structured interviews. Only one district, Mangaung Metro Municipality, was chosen for the CHWs interviews, due to its high number of CHWs. Interviews were conducted virtually for the LVCS due to their geographically dispersed locations, and face-to-face for the other two groups. The interview guide was reviewed and approved by an expert before its implementation. The interview guide contained questions about experience and behaviours, opinions and values, feelings, knowledge and understanding, and sensory observations (McNabb 2020). Interviews were in English for persons with visual impairment and LVCS, with Sesotho for CHWs’ responses, as they were not comfortable with English and requested Sesotho. The information was later translated into English by the first author. The interview questions were based on the objectives of the study: To explore challenges and community support of CHWs and LVCS in the Free State province for hard-to-reach persons with visual impairment. Each interview session lasted approximately 1 h. Data collection continued until saturation was reached (Rahimi & Khatooni 2024). Interviews were conducted by another expert, and the first author was present the entire time, taking notes and including key aspects in the analysis. Both have experience working with people with visual impairment. The research team possesses expertise in public health, disability and visual impairment. Matieho B. Mokhua, a PhD candidate in public health, has experience working with individuals with low vision and visual impairment. Similarly, Hlabje C. Masemola, also a PhD candidate in public health, has expertise in qualitative research and experience in the field of low vision. A telephone recording application was used to record all the interviews.

### Data analysis

Thematic analysis following Yin’s five-step framework outline, namely compiling, disassembling, reassembling, interpreting and concluding, was used to analyse the data (Yin 2016). The trustworthiness of the study was ensured by conducting a pilot study to refine the interview guide, ensuring credibility. Transferability was also ensured by providing an in-depth description of participants, including their behaviours and emotions, to allow readers to determine the applicability of the findings to other contexts.

## Discussion

### Challenges

#### Poor access to available resources

**FIGURE 1:**
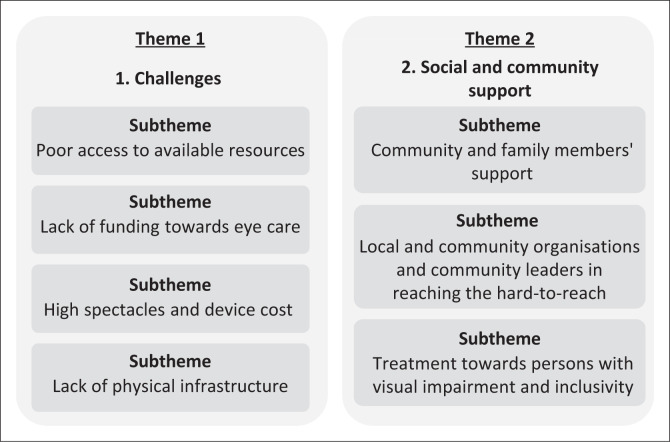
The two themes with their respective subthemes.

The findings reveal that resources that could be beneficial to persons with visual impairment from the local government structures and traditional leaders may be available to support them but are not utilised effectively by those responsible for them, creating barriers to service delivery (Figure 1). Findings also show inadequate support of the CHWs from the Department of Health, particularly in the provision of eye care services. Participants view these as factors that hinder their ability to meet community expectations and provide necessary healthcare interventions. Participants’ views and opinions are as follows:

‘We have a local government that is ruling there, and we also have chiefs ruling there, and what are they doing, because they have resources but it’s how they prefer to work that hinders the services to the relevant people.’ (VIP P2, Higher Certificate, 27 February 2025)

‘The Department of Health is failing us as CHWs a lot, because patients are expecting something from us and unfortunately majority do not have tools to even screen or check people with poor vision.’ (CHW P8, Grade 12, 14 February 2025)

The findings highlight a concerning lack of support for CHWs from the Department of Health, particularly concerning the provision of eye care services. Community health care workers show a strong willingness to address the needs of persons with visual impairment but are hindered by insufficient resources and training. Hence, the evidence indicates a lack of involvement of the CHWs in eye care services in South Africa (Xulu-Kasaba, Mashige & Naidoo 2021). Without the necessary tools, such as screening equipment, CHWs struggle to meet the expectations of the communities they serve. This lack of support not only impacts the effectiveness of CHWs, but also limits their capacity to deliver appropriate interventions, further exacerbating the gap in care for the hard-to-reach persons with visual impairment (Kyeremeh & Mashige 2021). Both findings indicate an underlying problem in the mobilisation of eye care services, where the lack of coordination between local leadership, health systems and community-based workers creates a barrier for the proper development of an eye service delivery model.

#### Cost of high spectacles and low-vision devices

The findings indicate that the affordability and accessibility of spectacles and low-vision devices remain a significant challenge. The same issue is raised by both LVCS and CHWs. According to participants, the cost challenges contribute to the low number of individuals in need, benefiting from these essential optical devices; participants’ views are reflected in the following statements:

‘If you could observe, you would find out that spectacles and eye devices are more expensive than other things that we get from other doctors.’ (CHW P8, Grade 12, 14 February 2025)

‘In my experience, problems in terms of devices’ cost remain a challenge.’ (LVC P3, Bachelor’s degree, 24 February 2025)

‘We are struggling to supply simple spectacles, so what about the low vision devices? That is why we see a very low number of persons with visual impairment seeking the services.’ (LVC P2, Bachelor’s degree, 24 February 2025)

Both LVCS and CHWs identified the high cost of the optical devices as a major obstacle that limits the number of individuals accessing or benefiting from them. Participants noted that the financial burden associated with acquiring spectacles and low-vision devices, coupled with the lack of funding for eye care services, restricts access to these vital resources, particularly in underserved and hard-to-reach communities. For the quality of life of persons with visual impairment to improve, low-vision devices and other means are also required. Evidence indicates that unaffordability can result in many individuals failing to benefit due to non-access to low-vision devices, further exacerbating their challenges (Sivakumar et al. 2020), making them hard-to-reach.

#### Lack of government funding towards eye care

There are significant barriers to accessing eye care services, particularly in rural areas. Persons with visual impairment feel neglected as they must travel to urban centres for care. Community clinics lack vision screening and eye care services, which contributes to the limited access to essential eye health interventions. The government is perceived to be failing to provide adequate funding for eye care, including the employment of optometrists and ophthalmologists, as well as the transportation of patients. Participants’ statements are as follows:

‘Urban areas are for those who are privileged to be here, that is why you see people from QwaQwa and other Xhariep sides coming here because they are forgotten.’ (VIP P2, Higher Certificate, 27 February 2025)

‘The government is failing to provide funds towards eye care.’ (CHW P8, Grade 12, 14 February 2025)

‘Eye care is expensive, so they do not want to release funds towards the employment of optometrists and eye doctors.’ (CHW P9, Grade 12, 14 February 2025)

‘You find out that there is underfunding and we are unable to execute our responsibilities optimally.’ (LVC P3, Master’s degree, 24 February 2025)

The findings of this study underline significant barriers that hinder the effective mobilisation of eye care services for persons with visual impairment in the Free State province, particularly in hard-to-reach areas. Available resources are not being fully utilised by local government structures. The underutilisation of resources can result from the ignorance of the existence of persons with visual impairment amongst the general community population (Ezeh et al. 2022). In theory, the collaboration between local health and non-local health organisations is known to improve patient outcomes (Alderwick et al. 2021). Thus, the lack of effective collaboration and resource mobilisation within these local leadership structures leaves many persons with visual impairment without adequate support and healthcare. Although there is minimal knowledge about the collaboration between local health and non-local health organisations in improving health outcomes, the expectation was that they could improve patient outcomes.

#### Lack of accessible physical infrastructure for persons with visual impairment

Persons with visual impairment face significant challenges related to mobility, which is compounded by the lack of accessible physical infrastructure in their environments. This makes it difficult for them to participate fully in daily activities and access necessary services. Some persons with visual impairment expressed their concerns as follows:

‘But the only problem that I think, actually, most of the visually impaired persons struggle with, is mobility and maybe a lack of physical infrastructure in their surroundings, that hinders their movement and their full potential.’ (VIP P1, Higher Certificate, 27 February 2025)

Poor infrastructure is another concern for persons with visual impairment. Prior evidence shows continuing challenges in their environment and their ability to navigate independently (Chidiac, Reda & Marjaba 2024). Additionally, addressing transportation challenges through subsidised or mobile eye care services could help reduce the geographic barriers that currently prevent many from receiving the care they need.

#### Community and family members’ support

Both urban and rural families and communities exhibit a low level of support for persons with visual impairment, which affects their well-being and access to resources. Persons with visual impairment are often not well supported, even by their immediate family members. The lack of proper support is evident when individuals with severe vision impairment are seen walking alone without proper assistive devices, highlighting the gaps in community and family-based care for them. Participants’ views and opinions are as follows:

‘For me, the community support from urban or rural seems to be at a very low level, and it affects both sides.’ (LVC P4, Master’s degree, 24 February 2025)

‘The visually impaired are not well taken care of by the communities, even by the immediate family members.’ (LVC P2, Master’s degree, 24 February 2025)

‘Somebody with that very poor vision walking alone, and that tells you that the visually impaired individuals, at times, are not well taken care of.’ (LVC P2, Bachelor’s degree, 24 February 2025)

Community health care workers further expressed that persons with visual impairment are often perceived as persons with mental impairment and incapable of performing basic tasks, which contributes to their marginalisation and social isolation. This perception, coupled with inadequate support and understanding of visual impairment from both the community and family members, perpetuates negative stereotypes. Without sufficient assistance from families and communities, individuals with visual impairment may face difficulties in performing everyday tasks, which can lead to increased isolation, dependence on others, and limited social connections, healthcare and economic opportunities. Studies indicate that persons with visual impairment continue to be negatively affected by loneliness and isolation more than the general population (Dunlop et al. 2024). The findings call for a shift in how families and communities view and care for persons with visual impairment, emphasising the importance of building a more inclusive, supportive and rights-based environment. Strengthening community-based care and providing families with the necessary tools and knowledge to care for persons with visual impairment are key components of mobilisation strategies that the local government should consider.

#### Inclusion, advocacy and community engagement with the hard-to-reach

Persons with visual impairment reiterate the importance of being actively involved in community programmes and decision-making processes, rather than being included only for public relations purposes. They advocate for the motto ‘nothing about us without us’ to be put into action, stressing the need for their direct involvement in initiatives that affect them. Community health care workers highlight the necessity of working closely with local leaders, such as councillors and pastors, to reach out to persons with visual impairment and to involve them in community activities. Below are the statements from some of the participants:

‘So I think the community leaders must involve us in their programmes; they don’t have to involve us only in public stunts or public relations issues, so it is we who will do justice to our sector.’ (VIP P3, Grade 12, 27 February 2025)

‘There’s a motto that persons with disability have which says “nothing about us without us,” and I think it’s time we put that into action.’ (VIP P2, Higher Certificate, 27 February 2025)

‘We want to be the access point, we fetch them and not the other way around.’ (CHW P8, Grade 12, 14 February 2025)

‘So, we must just involve the community and religious leaders, to spread the news.’ (LVC P2, Bachelor’s degree, 24 February 2025)

There have been advances over the past three decades in terms of our understanding of health knowledge in society, and health promotion that will remain relevant for the next 25 years (Edington et al. 2016). However, even with these advances, a lack of strong advocacy remains a challenge for the hard-to-reach in eye care in terms of its due prioritisation (Rao 2015). Mobilisation should prioritise the strategic use of available community and government resources, whilst ensuring the active inclusion of persons with visual impairment at all stages of community programme planning, decision-making and implementation (Munazza, Ahmed & Programme 2022). Engagements concerning community resources with local government structures and traditional leaders that remain underutilised should be prioritised. Community engagement in dialogues, awareness campaigns and structured information sharing by relevant institutions is vital in addressing misconceptions, reducing stigma and promoting an understanding of visual impairment.

#### Stigma and exclusion of persons with visual impairment, and the need for advocacy in disadvantaged communities

Community health care workers expressed concerns about persons with visual impairment often being excluded and treated poorly, particularly in disadvantaged communities. Despite these challenges, there has been some improvement in community awareness, though minimal. Some persons with visual impairment now feel safer and more confident in their communities, though they acknowledge that much more work in advocating for persons with visual impairment remains, especially for children. The need for continued advocacy and broader understanding of challenges experienced by persons with visual impairment is critical for further progress, and below are their views and opinions:

‘They are treated like mentally disabled people. Not treated well, and especially in our disadvantaged communities.’ (CHW P8, Grade 12, 14 February 2025)

‘Persons with visual impairments are not included in the decision-making and do not have support.’ (CHW P8, Grade 12, 14 February 2025)

‘We also fail to seek influence in participation.’ (LVC P2, Master’s degree, 24 February 2025)

‘We are now able to walk profoundly and confidently, and we feel a certain level of safety and feel that we are in our communities.’ (VIP P4, Bachelor’s degree, 27 February 2025)

‘I still believe that there’s still more work to be done, especially for the kids, because as an individual to be accommodated, I had to stand up for myself, so that’s why my community understands, but we have to consider the little kids.’ (VIP P4, Bachelor’s degree, 27 February 2025)

This improvement was achieved by raising awareness about visual impairment in communities. Incremental progress has been made in terms of social inclusion. However, this improvement is seen as insufficient, and participants emphasise that much more work remains, particularly when it comes to advocating for the needs of children with visual impairment. However, participants also reported having to teach the community themselves how they should be treated. Children still face challenges concerning the treatment they are getting from their communities. Evidence shows that these community improvement benefits are not equally available to all (Terras, Hendry & Jarret 2019).

## Conclusion

The lack of resources available for CHWs and LVCS to adequately support persons with visual impairment, the challenge of providing affordable eye care, poor infrastructure and the unaffordability of low-vision devices have become even more pronounced both at the community and professional levels. These challenges limit the capacity to deliver appropriate interventions to persons with visual impairment, further exacerbating the gap in care for the hard-to-reach. The situation emphasises a clear need for a more equitable distribution of resources, increased funding for eye care services, and the employment of more specialised professionals in underserved areas. Increased culturally sensitive dialogues and advocacy through faith-based and traditional platforms, in shaping positive narratives, reducing stigma and promoting inclusive values, are necessary in communities.

### Limitations

The LVCS’ interviews were virtual, and this may have had some limitations, preventing close interaction with participants. CHWs who were interviewed were from one district; therefore, the findings represent only one province, and resources may differ from one province to another. For the CHWs’ interviews, only one district in the province represented all five, and their challenges and experiences may be different.
